# Can Artificial Intelligence Create an Accurate Colonoscopy Bowel Preparation Prompt?

**DOI:** 10.1016/j.gastha.2024.10.006

**Published:** 2024-10-10

**Authors:** Marni H. Wilkoff, Nicholas R. Piniella, Rashmi Advani

**Affiliations:** 1 Department of Medicine, Icahn School of Medicine at Mount Sinai, New York, New York; 2 New York Institute of Technology College of Osteopathic Medicine, Glen Head, New York; 3 Division of Gastroenterology, Icahn School of Medicine at Mount Sinai, Mount Sinai South Nassau, One Healthy Way, Oceanside, New York

**Keywords:** Colon Cancer Screening, Adenoma Detection Rate, Machine Learning, Split-Dose Bowel Preparation, Colonoscopy Preparation

## Abstract

**Background and Aims:**

Colorectal cancer is the third most common cancer in the United States, with colonoscopy being the preferred screening method. Up to 25% of colonoscopies are associated with poor preparation which leads to prolonged procedure time, repeat colonoscopies, and decreased adenoma detection. Artificial intelligence (AI) is being increasingly used in medicine, assessing medical school exam questions, and writing medical reports. Its use in gastroenterology has been used to educate patients with cirrhosis and hepatocellular carcinoma, answer patient questions about colonoscopy and provide correct colonoscopy screening intervals, having the ability to augment the patient–provider relationship. This study aims at assessing the accuracy of a ChatGPT-generated precolonoscopy bowel preparation prompt.

**Methods:**

A nonrandomized cross-sectional study assessing the perceptions of an AI-generated colonoscopy preparation prompt was conducted in a large multisite quaternary health-care institution in the northeast United States. All practicing gastroenterologists in the health system were surveyed, with 208 having a valid email address and were included in the study. A Research Electronic Data Capture survey was then distributed to all participants and analyzed using descriptive statistics.

**Results:**

Overall, 91% of gastroenterologist physicians determined the prompt easy to understand, 95% thought the prompt was scientifically accurate and 66% were comfortable giving the prompt to their patients. Sixty four percent of reviewers correctly identified the ChatGPT-generated prompt, but only 32% were confident in their answer.

**Conclusion:**

The ability of ChatGPT to create a sufficient bowel preparation prompt highlights how physicians can incorporate AI into clinical practice to improve ease and efficiency of communication with patients when it comes to bowel preparation.

## Introduction

Colorectal cancer (CRC) is one of the most common cancers in the United States, accounting for nearly 10% of all cancers and 8% of all cancer-related deaths.^1^^,^^2^ It is usually diagnosed in individuals older than 50 years, but in recent years, the incidence in young adults has been steadily increasing, and now has become the third leading cause of cancer-related deaths among individuals younger than 50 years old.^3^ Colonoscopy is the most widely used method for CRC screening and has been shown to decrease CRC incidence and deaths through detection and removal of polyps, with the extent of bowel preparation being associated with the rate of adenoma detection.^4^^,^^5^ Optimal bowel preparation is required to fully visualize the colonic mucosa, though up to 25% of colonoscopies have poor bowel preparation.^4^, ^5^, ^6^ Inadequate bowel preparation can lead to procedure difficulty, prolonged procedure time, decreased adenoma detection, with some studies reporting a miss rate of up to 48%, frequent repeat colonoscopies, expensive costs, lower cecal intubation rate and high rates of electrocautery.^4^^,^^6^

Artificial intelligence (AI) is a rapidly evolving topic in medicine and has become widely used by gastroenterologists (GIs) to detect lesions, create risk stratification scoring systems, assess treatment response and create a bowel preparation scoring system.^7^ In 2022, OpenAI created Chat Generated Pre-Trained Transformer (ChatGPT), a type of large-language model, which has the potential to enhance patient education and support the relationship between the patient and provider.^8^^,^^9^ Recent studies have assessed ChatGPT’s ability to assess medical school exam questions, such as the United States Medical Licensing Exam, write medical reports, provide knowledge to patients with cirrhosis and hepatocellular carcinoma, answer patient questions about colonoscopy, and provide correct colonoscopy screening intervals.^8^^,^^10^, ^11^, ^12^, ^13^ The accuracy of ChatGPT-generated responses highlights the ability of large-language models to assist health-care professionals in making educated decisions regarding patient care and is a potential avenue for patients to receive succinct, easy to understand, reliable, and accurate information regarding medical management.^8^^,^^12^

Patient education is key for successful bowel preparation, and the use of ChatGPT can help bridge a gap, providing patients with easy-to-understand information to aid with the preparation.^6^^,^^8^^,^^14^ The American College of Gastroenterology emphasizes the use of split-dose preparation to ensure adequate bowel preparation and visualization during colonoscopy compared to whole dosing, improving patient satisfaction, compliance and adenoma detection rate.^6^ Since patient education and health literacy is instrumental to adequate bowel preparation, instructions should be adjusted to match their level of education.^6^ As more than one-third of the United States population has poor health literacy, colonoscopy bowel preparation instructions should be written at a sixth-grade reading level to decrease the potential of inadequate bowel preparation as instructions can be complex.^15^

ChatGPT not only has the benefit to improve patient understanding and access to reliable medical information, but has the potential to augment the physician and patient relationship. While studies have been performed investigating ChatGPT’s use in answering patient questions about colonoscopy and for postcolonoscopy management, there has not been a study assessing the accuracy of a ChatGPT-generated bowel preparation prompt.^10^^,^^11^ In this study, we aim to assess the scientific accuracy, comprehensibility, and comfort in using a ChatGPT-generated split-dose bowel preparation prompt in clinical practice.

## Methods

### Study Approval

This observational cross-sectional survey study was determined exempt by the Institutional Review Board at the Icahn School of Medicine at Mount Sinai in New York, NY, United States. Instructions for the survey included the agreement of informed consent upon completion and submission of the survey.

### ChatGPT and Response Generation

ChatGPT is a large language model that uses information from websites, online books and articles to generate easy to understand and accurate responses.^7^ Individuals can input a prompt and ChatGPT will respond using the information in its databank and has the capability to incorporate feedback and corrections provided by humans to improve responses.^7^ In this study, the following prompt was presented to ChatGPT (GPT-3.5): “Explain colonoscopy preparation to a patient using split-dose preparation.” The prompt was presented 3 times to ensure reproducibility. An online readability tool (readable.com) used validated reading-level metrics including the Flesch-Kincaid Grade Level and Gunning Fog Index to determine the reading level of the ChatGPT-generated response.

### Research Electronic Data Capture Survey

A 16-question Research Electronic Data Capture survey, a secure web-based application designed to support data collection and management, was created focusing on the ChatGPT-generated response. Information on respondent sex, whether the responder was a GI fellow or practicing gastroenterologist, years of experience, number of colonoscopies performed, and procedural practice setting was collected (Table 1). The ChatGPT-generated response was then presented and included follow-up questions asking the responder to rate the prompt for the following indicators on a 4-point Likert scale: comprehensibility, scientific accuracy, and comfort with distributing the prompt to patients (Table 2). Options for disagreement with the prompt included too much medical jargon, prompt being too long, confusing sequence of events, inaccurate dietary recommendations and timing of the second dose of bowel preparation, failure to mention medication recommendations or preprocedure clearance, no mention of when patient should take nothing by mouth, prompt is too generic, and the patient population would benefit from a different bowel preparation prompt. Responders were asked to determine if the prompt was better than the one currently used in their practice (Table 3). Responders were also asked to interpret if the prompt was generated by AI or a human, and how confident they were with their answer (Table 4).

**Table 1:** Demographics Table

Characteristics: no. (%)	Fellow (n = 14)	Practicing gastroenterologist (n = 30)
Sex		
Male	9 (64)	21 (70)
Female	5 (46)	9 (30)
Years of experience		
PGY4 fellow	4 (29)	··
PGY5 fellow	5 (36)	··
PGY6 fellow	5 (36)	··
<10 y	··	15 (30)
>10 y	··	15 (50)
Number of colonoscopies performed		
<100	4 (29)	0
100–500	7 (50)	2 (7)
500–1000	3 (21)	3 (10)
1000–1500	0	3 (10)
1500–2000	0	1 (3)
>2000	0	21 (70)
Procedural practice setting		
Community hospital	2 (14)	3 (10)
Academic hospital	8 (57)	11 (37)
Ambulatory surgical unit	0	6 (20)
Outpatient office with endoscopy suite	0	3 (10)
Multiple sites	4 (29)	7 (23)

PGY, post-graduate year.

**Table 2:** Assessment of Quality Indicators by Years of Experience

Years of experience	Participants who agreed with the following statements: no. (%)		
	*“The prompt is easy to understand”*	*“The prompt is scientifically accurate”*	*“I am comfortable giving this prompt to my patients”*
Total (n = 44)	40 (91)	42 (95)	29 (66)
Fellow (n = 14)	14 (100)	14 (100)	11 (79)
First y (n = 4)	4 (100)	4 (100)	4 (100)
Second y (n = 5)	5 (100)	5 (100)	3 (60)
Third y (n = 5)	5 (100)	5 (100)	4 (80)
Practicing gastroenterologist (n = 30)	26 (87)	28 (93)	18 (60)
<10 y (n = 15)	13 (87)	13 (87)	10 (67)
>10 y (n = 15)	13 (87)	15 (100)	8 (53)

**Table 3:** Comparison to Prompt Used in Current Practice

Years of experience	Perceptions of the prompt: no. (%)		
	Same/Better	Worse	*P* value
Total (n = 44)	30 (68)	14 (32)	··
Fellow (n = 14)	11 (79)	3 (21)	.49
Practicing gastroenterologist (n = 30)	19 (63)	11 (37)	
<10 y (n = 15)	9 (60)	6 (40)	1.0
>10 y (n = 15)	10 (67)	5 (33)

**Table 4:** AI- or Human-Generated Prompt

Years of experience	AI: no. (%)	Human: no. (%)	*P* value
Total (n = 44)	28 (64)	16 (36)	··
Fellow (n = 14)	10 (71)	4 (29)	.46
Practicing gastroenterologist (n = 30)	18 (60)	12 (40)	
<10 y (n = 15)	11 (73)	4 (27)	.14
>10 y (n = 15)	7 (47)	8 (53)	

All practicing gastroenterologists and GI fellows within a large quaternary-care health system were included in the study. Contact information was obtained from the health system website. Of the names obtained, 73 were excluded as no valid email address was associated with the individual. A total of 208 physicians were included in the study. As this is a qualitative analysis, sample size is difficult to calculate as there is no formula; however, these 2 statistical methods were used: “purposive sampling” and “saturation.” Purposive sampling of the research question was performed as it was geared specifically toward gastroenterologists at different stages of their career and thus allowed for the results to be representative of the cohort being studied. The results therefore equally reflect samples from each cohort of gastroenterologists studied. Saturation of the results was reached which was accounted for in the appropriate sample size value of 23. Continuous analysis of the data was also performed. Twenty one percent of reviewers responded to the survey.

### Outcomes and Statistical Analysis

The primary outcomes of this study include scientific accuracy, understandability, and comfort distributing the prompt to patients. Secondary outcomes include if the prompt was the same or better than the current prompt used in their practice and determination if the prompt was generated by AI or human. All analyses were conducted using JASP version 0.16 (University of Amsterdam, Amsterdam, The Netherlands). Descriptive statistics were used to compare demographic and accuracy, ease of understanding and comfort distributing the prompt, as numbers with proportions. Fisher’s exact test was used to analyze the usability of the prompt. Statistical significance was evaluated at *P* < .05.

## Results

The response rate was 21.2% (44 of 208). Thirty practicing gastroenterologists (68%) and 14 GI fellows (32%) completed the survey (Table 1). The full ChatGPT-generated response scored a 9.0 on the Flesch-Kincaid Grade Level and 15.7 on the Gunning Fog Index and can be found in the Supplementary Material.

Ninety one percent of gastroenterologists (40 of 44) felt the response was easy to understand, 95% (42 of 44) felt it was scientifically accurate, and 66% (29 of 44) were comfortable distributing the prompt to their patients (Table 2). One hundred percent of GI fellows (14 of 14) and 87% of practicing gastroenterologists (26 of 30) felt the response was easy to understand (*P* = .29), 100% of GI fellows (14 of 14) and 93% practicing gastroenterologists (28 of 30) felt the response was scientifically accurate (*P* = 1.0), and 79% of GI fellows (11 of 14) and 60% of practicing gastroenterologists (18 of 30) were comfortable distributing the prompt to their patients (*P* = .31) (Table 2).

Sixteen practicing gastroenterologists, 2 second-year fellows, and 1 third-year fellow disagreed with various aspects of the prompt (Figure). Common reasons for disagreement include the response being too generic and long, no mention of nothing by mouth status or dietary and medication recommendations (Figure).

**Figure:**
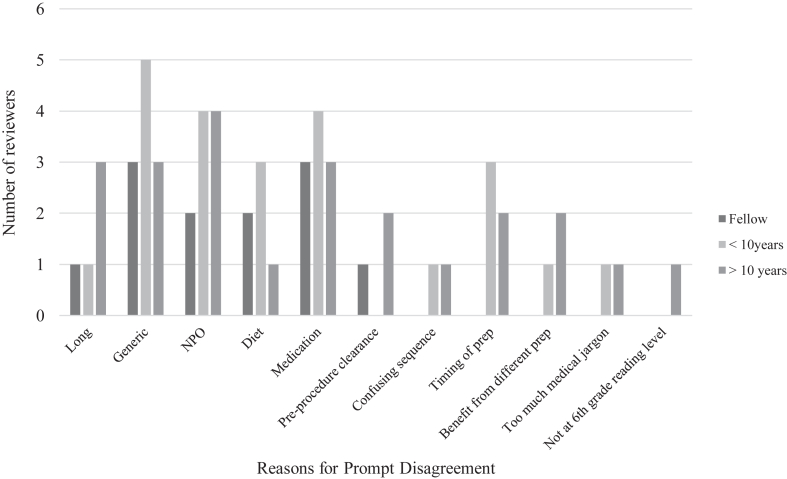
Distribution of reasons for disagreement with the prompt broken down into fellows and practicing gastroenterologist with more than 10 years and less than 10 years of experience. Reasons for disagreement include the prompt being too long, prompt is too generic, no mention of NPO status, poor diet recommendations, no mention of medication specifications, no mention of preprocedure clearance, confusing sequence of events, inaccurate timing of the bowel preparation, too much medical jargon, and that the prompt is not at a sixth-grade reading level. NPO, nothing by mouth.

Reviewers demonstrated 64% accuracy in identifying the AI-generated answer, but only 32% (9 of 28) were confident in their decision (Table 4). Sixty percent (18 of 30) of practicing gastroenterologists thought the prompt was generated by AI, but only 39% were confident in their answer (Table 4). Fifty percent of the first-year fellows (2 of 4), 100% of the second-year fellows (5 of 5), and 60% of the third-year fellows (3 of 5) thought the prompt was generated by AI, with 20% being confident in their decision (Table 4).

## Discussion

This is the first study in the literature examining the use of ChatGPT in creating a scientifically accurate and easy to understand colonoscopy bowel preparation prompt. The results show that ChatGPT can create a bowel preparation prompt that most of the evaluators agreed with and were comfortable distributing to their patients; however, we also highlighted that there is some room for improvement for AI-generated responses to address concerns that were felt by more senior gastroenterologists.

*Lee et al*. determined that ChatGPT provided easy to understand, scientifically accurate and satisfactory answers to common patient questions about colonoscopy.^10^*Gorelick et al*. found that ChatGPT had 90% compliance and 85% accuracy with postcolonoscopy surveillance management.^12^ Furthermore, *Lim et al*. determined that providing ChatGPT with contextualized medical guidelines allows it to provide accurate colonoscopy screening intervals.^11^ Our study builds off the current literature and uses ChatGPT to create a bowel preparation prompt.

This study included both GI fellows and practicing gastroenterologists with a broad range of experience, allowing for 2 distinct groups to review the prompt. The GI fellows had a greater overall agreement with the prompt compared to practicing gastroenterologists, highlighting that those with more experience were less comfortable distributing the prompt to their patients. Those who disagreed with the prompt deemed it too generic, long, medically complex, and contained inadequate dietary and medication recommendations. While there was some disagreement with the prompt, there was an overall positive reaction, suggesting that ChatGPT can generate an acceptable bowel preparation prompt that can be modified to address these issues.

The response was graded by online readability tools and was determined to be above the recommended sixth-grade reading level determined by the American Gastroenterological Association.^15^ The readability of 2 bowel preparation prompts used within our health system were found to be more advanced than the recommended sixth-grade reading level, scoring an 8.5 and 7.8 on the Flesch-Kincaid Reading Level and 10.5 and 8.6 on the Gunning Fog Index. When comparing these to the AI-generated prompt, the AI prompt was found to be more complex than the current prompts used in practice. As more than one-third of people in the United States have poor health literacy, these findings highlight a barrier for a large population and emphasize the importance of tailoring bowel preparation prompts to a certain level of education.^15^ To ensure the prompt is appropriate for various education levels, it is important to modify it, allowing all patients to understand the instructions. Having instructions that are too advanced for an individual can lead to poor bowel preparation compliance and success.^5^ Creating multiple versions of bowel preparation instructions has the potential to create more work for physicians, but with the help of ChatGPT, one can query the large language model to customize the response to a specific education level.

Overall, reviewers were able to detect the ChatGPT-generated prompt, fellows more so than practicing gastroenterologists, though were not confident in their decision. This could be due to fellows having more experience with advanced technology during their training. We used the separation between fellows and attendings by year of experience as a proxy for age, to see if younger physicians were more likely to think the response was AI-generated.

Strengths of the study include this being the first study of its kind evaluating the use of ChatGPT in creating a bowel preparation prompt and the findings add valuable information on the use of AI in gastroenterology (Table 5). Data were also analyzed by using length of time in practice as a proxy for age to evaluate if younger physicians were more likely to think the prompt was generated by AI (Table 5). Limitations of the study size include a small sample size of 208 gastroenterologists with a 21% response rate (Table 5). The study was geographically confined to 1 health-care system in the northeast, and it was a nonrandomized cross-sectional analysis (Table 5).

**Table 5:** Strengths and Weaknesses of the Study

Strengths	Limitations
First study evaluating this topic	Small sample size
Data analysis stratified by length of time in practice	Geographically limited to one health-care system
Provide valuable information on the use of AI in GI	Nonrandomized, cross-sectional analysis

The current AI-generated bowel preparation prompt is adequate but requires more work and review to comfortably disseminate to patients. Providing ChatGPT with contextualized medical information can help further expand on areas that are considered vague. In addition to using ChatGPT to incorporate contextual knowledge, an experienced gastroenterologist should review and provide additional expertise. While improvements to the prompt should be made, it is important to ensure that the reading level is appropriate for all patients. Using ChatGPT to assist with creation of bowel preparation prompts can enhance ease and efficiency of communication between physicians and patients and can potentially highlight another avenue for patients to seek information.

## Figures and Tables

**Figure A1:**
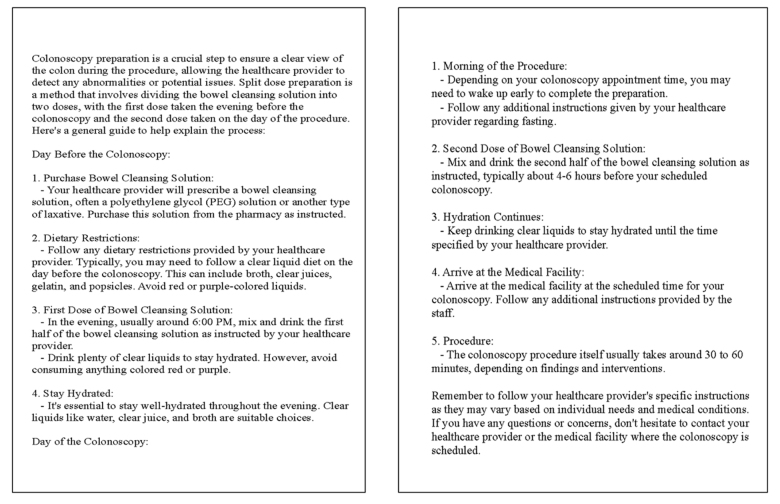
The full ChatGPT-generated prompt can be seen here.
