# Supplementary material for: Can Artificial Intelligence Create an Accurate Colonoscopy Bowel Preparation Prompt?
Source: Gastro Hep Adv. 2024 Oct 10;4(2):100566. doi: 10.1016/j.gastha.2024.10.006 (PMC11758412; doi:10.1016/j.gastha.2024.10.006)
Supplement: Supplemental Materials [file mmc1.docx]

Colonoscopy preparation is a crucial step to ensure a clear view of the colon during the procedure, allowing the healthcare provider to detect any abnormalities or potential issues. Split dose preparation is a method that involves dividing the bowel cleansing solution into two doses, with the first dose taken the evening before the colonoscopy and the second dose taken on the day of the procedure. Here's a general guide to help explain the process:

Day Before the Colonoscopy:

1. Purchase Bowel Cleansing Solution:

- Your healthcare provider will prescribe a bowel cleansing solution, often a polyethylene glycol (PEG) solution or another type of laxative. Purchase this solution from the pharmacy as instructed.

2. Dietary Restrictions:

- Follow any dietary restrictions provided by your healthcare provider. Typically, you may need to follow a clear liquid diet on the day before the colonoscopy. This can include broth, clear juices, gelatin, and popsicles. Avoid red or purple-colored liquids.

3. First Dose of Bowel Cleansing Solution:

- In the evening, usually around 6:00 PM, mix and drink the first half of the bowel cleansing solution as instructed by your healthcare provider.

- Drink plenty of clear liquids to stay hydrated. However, avoid consuming anything colored red or purple.

4. Stay Hydrated:

- It's essential to stay well-hydrated throughout the evening. Clear liquids like water, clear juice, and broth are suitable choices.

Day of the Colonoscopy:

1. Morning of the Procedure:

- Depending on your colonoscopy appointment time, you may need to wake up early to complete the preparation.

- Follow any additional instructions given by your healthcare provider regarding fasting.

2. Second Dose of Bowel Cleansing Solution:

- Mix and drink the second half of the bowel cleansing solution as instructed, typically about 4-6 hours before your scheduled colonoscopy.

3. Hydration Continues:

- Keep drinking clear liquids to stay hydrated until the time specified by your healthcare provider.

4. Arrive at the Medical Facility:

- Arrive at the medical facility at the scheduled time for your colonoscopy. Follow any additional instructions provided by the staff.

5. Procedure:

- The colonoscopy procedure itself usually takes around 30 to 60 minutes, depending on findings and interventions.

Remember to follow your healthcare provider's specific instructions as they may vary based on individual needs and medical conditions. If you have any questions or concerns, don't hesitate to contact your healthcare provider or the medical facility where the colonoscopy is scheduled.
